# Neural Predictors of Changes in Party Closeness after Exposure to Corruption Messages: An fMRI Study

**DOI:** 10.3390/brainsci11020158

**Published:** 2021-01-26

**Authors:** Juan Sánchez-Fernández, Luis-Alberto Casado-Aranda

**Affiliations:** Department of Marketing and Market Research, University of Granada, 18010 Granada, Spain; sanchezf@ugr.es

**Keywords:** consumer neuroscience, corruption advertising, political behavior, party closeness, psychological mechanisms, neuroimaging

## Abstract

Daily worldwide newspapers are filled with campaigning unveiling political corruption. Despite this information be worrying to many citizens, political researchers have not identified any consistent trend of decline of support among party sympathizers. This study utilizes neuroimaging for the first time to examine the neuropsychological origin of party closeness variation among backers of a liberal (Spanish Socialist Workers’ Party, PSOE) and a conservative party (Popular Party, PP) in Spain after a month receiving corruption messages among their preferred party. Brain data provide some explanation as to the origin of party closeness reduction among liberal sympathizers: areas involved with negative feelings, disappointment and self-relevance served to predict party closeness reduction 30 days in advance. Implications for liberals and conservatives’ campaigns are discussed.

## 1. Introduction

Corruption continues to be a challenge for the world and specifically for Europe. It is a phenomenon that costs the European economy between €179 billion and €950 billion each year. Daily worldwide newspapers are filled with articles unveiling political corruption, which takes many forms: trading in influence, bribery, conflicts of interest or nepotism [1]. However, do these political messages drive to a loss of closeness, sympathy or votes for the corrupt parties?

### 1.1. Political Communication Campaigns

The findings of certain research in the field of advertising have strongly backed the importance of the role of messages revealing actions of political parties in shaping perceptions and closeness to both politicians and parties, and their influence on voting behavior [2,3]. Merritt [4] or Pattie et al. [5], for instance, have identified that negative political communications (messages seeking to demean the perception of an opposite party or candidate) evoke negative effects toward both the targeted opponent and the sponsor. Roddy and Garramone [6] reported that although voters evaluate a negative-response communication less favorably than a positive one, the first was more efficient in reducing voting for the attacking candidate. Lau et al. [7,8] concluded that negative campaigning does reduce perceived trust in government, political efficacy and overall citizenship feelings. Along the same line, previous research suggests that one type of negative messages, namely those related to corruption—the focus of the current paper—undermine voter confidence in public institutions [9,10], reduce trust in politicians [11] and lower voter confidence in government and candidate ability [12]. Recent research goes even further and suggests that information about own party political corruption leads to the loss of votes and to a decrease in identification and sympathy [13]. Krupnikov et al. [14] concludes, furthermore, that negative campaign communication directly influences the willingness of an individual to make a candidate selection. Along the same line, Erlingsson et al. [15] assessed the way in which 2008’s international crisis affected the relevance of perceptions of corruption in that period in Iceland. The authors concluded that political support decreased after the crisis and citizenship assessments of corruption became the most remarkable variable of support. More recently, Vera [16] evaluated how candidate competence in public works and corruption prevalence affect electoral accountability for corruption. The findings show that even typologies of corruption with side benefits would be roughly when related to unskilled politicians.

Yet the findings of other analyses differ and conclude that voters do not unseat corrupt politicians. These studies demonstrate, against the rational precepts of democratic theory and the traditional models of decision-making [17], that electoral punishment of political corruption is not a reality and citizens do not punish corruption when it proves more competent than honest leaders. This is the case of the analysis of Rennó [18] that determined that voter worry about corruption had weak incidence on voting behavior in the presidential election of Brazil in 2006 as the electorate simply cast their ballot for their preferred candidate. Furthermore, candidates for the US House of Representatives charged with corruption, according to Peters and Welch [19] do not suffer major citizenship disapproval and are likely to be re-elected. Similarly, Martinez and Delegal [20] concluded that participants viewing strong negative messages reported comparable levels of trust in the government as those evaluating a less negative campaign. Along the same line, the study by Rivero and Fernández-Vázquez [21] claims that parties of cities in Spain, the country of the current analysis, whose mayors are involved in corruption scandals suffered no consequences at the ballot box, especially among their own sympathizers [22]. Barros et al. [23] also corroborated that the Brazilian vote for corrupt candidates despite having information that their representatives have been related to corruption. According to those authors, ideology plays a key role in explaining this voting behavior.

### 1.2. Reasons Underlying the Effects of Political Messaging: Partisanship and Psychological Mechanisms

Two main determinants throw light on the lack of consensus as to the effects of advertising unveiling corruption on the decrease of party closeness and voting behavior. Firstly, literature on political behavior does conclude that sympathizers of specific parties tend to de-emphasize corruption practices referred to their own party [24]. This so-called “partisan reasoning” takes place when inhabitants assess facts in ways that allow them to control or even bolster their attitudes in the face of contradictory evidence, such as corruption messages linked to their party of preference [25]. Previous studies exploring information processing do confirm that people who hold steady thoughts on complex social issues (such as political opinions) are likely to evaluate remarkable empirical evidence in a biased manner. Specifically, they are apt to accept “confirming” evidence at face value while subjecting “discontinuing” evidence to critical evaluation, and as a result to draw undue support because of their initial thoughts from random empirical messages [26,27].

In the field of political behavior, specifically, partisan bias can affect the processing of corruption messages referring to their own party. Indeed, Eggers [28] confirms that attitudes towards political corruption are mediated by partisanship. Yet such partisan bias may affect individuals of liberal (left) and conservative (right) parties differently, previous research coincided on their differences on cognitive and motivational functioning. For example, Jost et al. [29] concluded that conservative individuals are more patriotic and tolerant of inequality, while liberal voters are more autocritical and intolerant of own party corruption. Lining up with this reasoning, the theory of cognitive dissonance explains that unpleasant psychological states may result from inconsistency between two or more elements in a cognitive system [30]. Prior studies at this regard found that conservatives (vs. liberals) judge attitude-inconsistent news messages (e.g., corruption messages about their preferred political party) as less credible than attitude-consistent news sources, as they experience higher cognitive dissonance [31]. This psychological process could lead to conservative sympathizers not reducing their sympathy for their parties because of disbelief in the corruption associated with them. Previous studies in the field of political behavior psychology confirm that conservatives possess greater self-esteem, ego and confidence in their political party than liberal citizens [32,33]. Sympathizers supporting these two parties may also differ in terms of six evolutionary key traits: care, fairness, loyalty, authority, sanctity and liberty as proposed by Jonathan Haidt [34]. Specifically, political psychology literature has shown that the left-supporters rely primarily on care and fairness while the right-sympathizers rely also on loyalty, authority, sanctity and liberty. The absence of the values of superiority and loyalty in the liberal party could explain that the liberals reduce their sympathy for their party to a greater extent than the conservatives. The work by Schreiber et al. [35] even showed that the brain structure of liberals and conservatives is different and they experience disparate cognitive processes when they think about risk. Accordingly, it is reasonable to assume that the firm closeness of sympathizers of the conservative (right) to their own party may not be as affected by the unveiling of this information as that of liberal (left) voters exerted on parties of their inclination. Two papers approach and back up such reasoning. The study of de Figueiredo et al. [36] concluded that exposing the criminal record of center-right candidates, in fact, had no effect on the vote of sympathizers. Riera et al. [37] found that politically-involved liberal voters of the PSOE more actively punished both liberal and conservative corrupt politicians.

Secondly, it is conceivable that there are implicit processes that take place during exposure to information about corruption that remain unreachable to conscious awareness, or conscious processes that are hard to be captured with a summary ratings/surveys following reception of the messages that might provoke a change or immobilism of party closeness more objectively than biased self-report methods [38]. Indeed, recent studies in the field of political behavior are beginning to use more objective and precise techniques from neuroscience to gain a deeper insight into the audience decision-making processes in political contexts [39,40].

### 1.3. The Current Study

This study constitutes a first step to address these research gaps from the perspective of neuroimaging. Following similar studies in other fields of social science, we contend that neuroscience can help identify the psychological mechanisms underlying the perception of corruption messages as a function of political ideology. The current research focuses on corruption advertising, and not policy failures through incompetence, for example, because of two reasons: (i) there exists a recently emerging literature on the effects of media coverage of corruption on citizens perceptions and behaviors (Kim and Baek 2018) and (ii) information about corruption is considered highly emotional, engaging and impactful, hence exploring its psychological (neural) mechanisms constitutes a crucial step to understand how political corruption messages affect citizens. The large amount of evidence showing that the partisan reasoning exerts the greatest influence when subjects confronting to contradictory information [41,42,43,44] supports also the use of messages about corruption referred to the preferred party in the current research.

This investigation particularly explores the neural origin of alteration of party closeness among sympathizers of conservative and liberal parties in response to advertising exposing corruption of their own party. We specifically aim to reveal which brain areas show increased activation in response to these messages so as to predict party closeness change after a month of real intensive-communication campaigns exposing corruption of the parties supported by the participants. The study is limited to supporters of the two main parties in Spain, the more liberal PSOE and the more conservative PP. It is noteworthy that the number of Spanish notorious corruption scandals in the realm of the political arena has surged in recent years [45]. Delving deeper into the neuroimaging processes triggered by information pinpointing corruption among liberal and conservative parties not only serves to examine ideological asymmetries between sympathizers in cognitive and motivational functioning, but is essential to advance the understanding of the psychological mechanisms by which political advertising affects political affiliation or even changes in voter behavior.

This study therefore focused on measuring neural activity in a series of a priori brain regions of interest (ROI) typically associated with the negativity, risk or distrust that corruption messages can evoke to sympathizers of a political party [7,12]. More specifically, we expect the activations of the amygdala, anterior insula, striatum and caudate, while exposing the participants to information revealing corruption linked to their preferred political party. The data gleaned from the measurements would be then correlated with the values of change of party closeness among the individuals after exposure to corruption of their own party during a one-month period. The choice of examining these neural regions is based on both theoretical and data-driven grounds. The amygdala is traditionally linked to the penalty domain [46], untrustworthy environments [47], fear and negative emotions [48]. Interestingly, the functional Magnetic Resonance Imaging (fMRI) study by Westen [44] reported for the first time the neural correlates explaining the motivated reasoning in the political context and found activity in the left portion of the amygdala during the evaluation of incongruent/threatening messages about a preferred political candidate also identified. Moreover, the anterior insula was persistently activated across a host of aspects including disappointment [49], arousal [50] and emotional processes [46]. Despite the fact that the striatum constitutes a key brain area in the dopaminergic reward system, it simultaneously engages negative effects in the penalty domain [46]. A vast amount of research has also identified the caudate as a brain region involved in negative and risky decisions [51]. Furthermore, these regions have been shown to be involved with punishment and attitude change [52].

Accordingly, we propose that the elicitation of these brain regions during the exposure to supporters of corruption among their own party are associated with changes in closeness after the intensive one-month campaign of messages and even will serve to predict such behavioral modifications. Furthermore, it would be reasonable to expect such an effect to be more evident between liberal sympathizers, more autocritical and intolerant than their conservative opposites [29].

## 2. Materials and Methods

The Materials and Methods should be described with sufficient details to allow others to replicate and build on the published results. Please note that the publication of your manuscript implicates that you must make all materials, data, computer code and protocols associated with the publication available to readers. Please disclose at the submission stage any restrictions on the availability of materials or information. New methods and protocols should be described in detail while well-established methods can be briefly described and appropriately cited.

### 2.1. Participants

Thirty participants were recruited for the fMRI test. Each met the usual requirements for fMRI scanning: no metals in the body, no history of psychiatric care (as mental disorders could affect the neural mechanisms of interest in an unwanted way [53]) and no pregnancy. A detailed checklist of the medical requirements for participants can be found in the Appendix A. Before enrolling in the study, all filled out an informed consent form in line with both the Helsinki declaration and the Ethics Committee of Human Research of the University of X. The characteristics of fMRI studies (e.g., cost, time and accessibility) have led to reducing the sample size of these types of investigations [54]. In the field of social neuroscience, current impactful investigations using neuroimaging tend to use small sample sizes (from 10 to 30 participants) to predict behaviors, namely [55] or [56].

As the main goal of the research is to evaluate the differences among the neural predictors of party closeness change among PSOE and PP sympathizers when faced with messages exposing political corruption, the experiment only retained subjects whose ideologies aligned themselves with the two major Spanish political parties. The selection process therefore consisted of asking participants to respond to the following question: “To which of the following parties do you feel more sympathy or which party do you consider closer to your own ideas?” (1 = extremely far and 10 = extremely close) (“CIS·Centro de Investigaciones Sociológicas·Ficha del estudio” 2018). The average proximity of the 10 selected (5 male and 5 female) PP sympathizers was 8.80 (SD: 1.31) and the average proximity of the 10 (5 male and 5 female) PSOE supporters was 8.60 (SD: 1.08). No differences were identified between the two groups as to party closeness (Z (9) = 0.318; *p* = 0.76).

### 2.2. Procedure

Participants arrived an hour prior to the experiment. Their first task was to confirm the data of the informed consent form before taking part in an inspection to assure they fulfilled all the medical requirements. On day 1 of the experiment, before the scanning session, all subjects made declarations, once again, of their party proximity to verify if they serve as proxies of PP and PSOE sympathizers.

Next, during the fMRI scanning, subjects viewed 20 messages exposing corruption based on real news items linked to their party of preference (PP or PSOE). These messages were accompanied by party logos. Since fMRI studies require a certain amount of repetition to obtain statistically significant brain activations similar to multi-item scales, subjects were exposed three times to similar, albeit not identical, messages (Dimoka, 2010). Examples of these messages are “Deviation of public aid”, “Delivery of false diplomas” or “Illicit enrichment”. Prior to the session, subjects were requested to read along silently, view each slide attentively, and were informed that they would later be asked a series of questions regarding the slides (aiming to raise participant attention). Special attention was taken in the choice of messages to ensure a control of their complexity and length (between 3 and 5 words). In a pretest, an independent sample (*n* = 60) substantiated that all messages exposing corruption were perceived with similar levels of concern (7-Likert scale with 1 = not alarming and 7 = very alarming; mean = 6.17; SD = 0.84).

**Figure 1:**
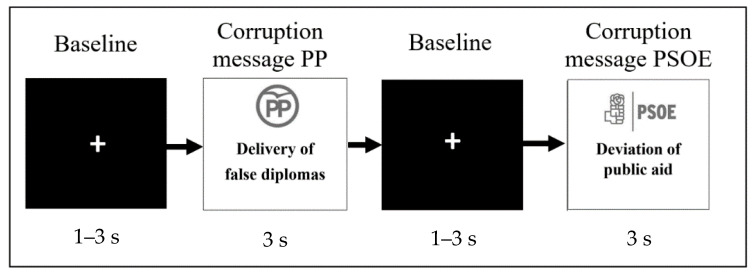
Depiction of the experimental design of the functional Magnetic Resonance Imaging (fMRI) task.

Each series of 20 messages began with a short (1–3 s) fixation cross display followed by a 3 s message accompanied by a logo of the participants own political party (Figure 1). The order of the messages and logos was counterbalanced among the subjects so that sympathizers of each political inclination view random messages exposing corruption of their party. Then fMRI material lasting about 5 min was shown via E-Prime Professional 2.0. The timing of each trial was similar to preceding fMRI experiments (Casado-Aranda et al., 2018). Participants were unaware on day 1 of the gathering of measurements of their level of party closeness. During the month after the scanning session, participants received four website links a week with references to real news, including items about corruption of their party of preference. Then they were asked to report feedback to ensure they had read the news. They were then contacted via e-mail to report on their party closeness by filling out the same scale they completed during the recruitment phase [57].

### 2.3. Data Acquisition and Analysis

#### 2.3.1. Behavioral Data

The level of change of party closeness was calculated subtracting the day 30 postscan value from the prescan party closeness value that the participant indicated having during the postscan month. The authors asked questions about the corruption advertisements to assure that participants read them.

#### 2.3.2. fMRI Data Acquisition Parameters and Analysis

The neural images of the different participants were recorded with a 3 Trio Siemens MRI Scanner. Images serving to judge the performance of the public/private sector comprised 500 volumes. A T2*-weighted EPI sequence (TR = 2000 ms, TE = 25 ms, FA = 90°, 35 slices and slice thickness = 3.5 mm) served to record the functional images. The structural image T1 made use of a sagittal orientation and a voxel size of 1 mm^3^ for coregistration and normalization.

The data gleaned from the fMRI scanning was analyzed by means of the Statistical Parametric Mapping software (SPM12, Wellcome Department of Cognitive Neurology, Institute of Neurology, London, UK) scripted with MATLAB R2012a (The Mathworks Inc., Natick, MA, USA). The functional images were realigned to the first image of the time series. The coregistration process was divided into two phases: in-plane T1 images were registered to the mean functional image while the high-resolution T1 images were registered to the in-plane image. High-resolution structural images were then normalized (retaining 3.5 mm × 3.5 mm × 3.5 mm voxels) according to the template of the Montreal Neurological Institute (MNI). A Gaussian kernel (7 mm FWHM) served to smooth the functional images.

A general linear model (GLM) for each subject was then generated with two regressors of interest: (i) onset slide including messages related to PSOE corruption (C_PSOE) and (ii) onset slide including messages related to PP corruption (C_PP). Moreover, each GLM included a constant session term, six covariates to capture residual movement-related artifacts, and fixation crosses as regressors of no interest. Images were realigned to correct for motion, normalized into standard stereotactic space, and smoothed with a 7 mm Gaussian kernel full-width half-maximum. We then modeled the task at the single subject level, comparing activity while receiving corruption messages referred to their preferred party to activity at rest. A random effects model was constructed, averaging over these single subject results at the group level.

To explore the brain regions where corruption messages about the participant’s party’s activation varied with participant differences in party closeness change, the contrast image of (i) C_PSOE or (ii) C_PP, respectively, were entered into a one-sample *t*-test in the second level random effect analyses with as the covariate the subtraction of the closeness party difference prescan and post30. ROI analyses were developed using small volume correction (SVC) as implemented in SPM. The use of SVC allows researchers to conduct principled correction resorting to the Gaussian random field theory within a predefined region of interest [58]. Particularly, we designed a mask containing spheres measuring 10 mm in radius based on coordinates obtained from previous studies analyzing the processing of negativity, risk or distrust. Specifically, to construct the amygdala ROI, amygdala coordinates related to the processing of incongruent messages were considered on the midline at the y and z coordinates reported by [44] (−22, −4, −12). To construct the anterior insula ROI associated with disappointment, we used the anterior insula coordinates reported by [49] (−30, 3, 9). To construct the anterior striatum ROI, the same procedure was followed based on the striatum coordinate reported by [46] (−16, 4, −4), which was associated with emotional negative processing. The same procedure was finally followed to construct the caudate ROI, according to the coordinates reported by [51], which linked this area to risky and negative processing.

To further assess whether neural activity in the a priori ROIs were also predictive of the sympathizer’s closeness party change after receiving 30 days of corruption messages, a linear regression analysis was applied using IBM SPSS Statistics Version 22 (SPSS) with a change in party closeness as the dependent variable and parameter estimates of the ROIs striatum, amygdala, anterior insula and caudate as independent variables.

## 3. Results

This section may be divided by subheadings. It should provide a concise and precise description of the experimental results, their interpretation and the experimental conclusions that can be drawn.

### 3.1. Behavioral Data

Statistical analyses were carried out with the IBM SPSS Version 20. Two paired sample *t*-tests (Wilcoxon because of the sample size) were created to evaluate whether there were significant differences in the levels of party closeness at the moment of the prescan and after the month of exposure to messages exposing corruption. The results indicate a significant decrease among PSOE sympathizers of party closeness after exposure to news items describing corruption during the month following the scan (Mprescan = 8.6, M post30 = 7.3, t (9) = 2.32; *p* = 0.04)). PP sympathizers, in turn, showed a constant level of party closeness after exposure to news indicating corruption among their party (Mprescan = 8.8, M post30 = 8.4, t (9) = 1.30; *p* = 0.22).

### 3.2. Brain Data

**Figure 2:**
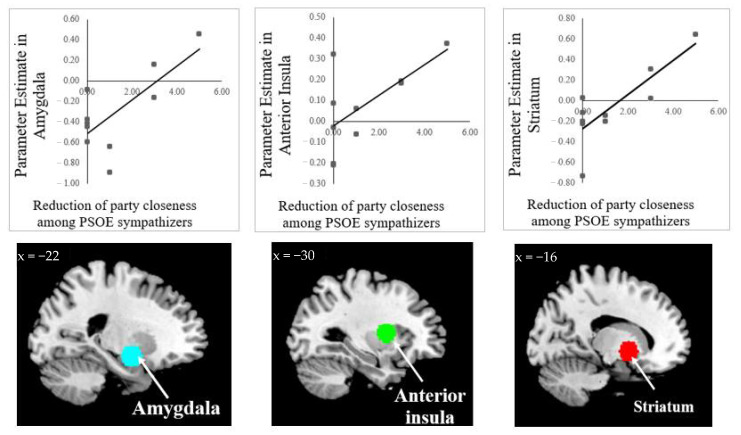
Illustration of the regions of interest taken as 10-mm spheres and their correlation to changes of party closeness. (Blue): amygdala (Westen et al., associated with incongruent processing [44]); (Green): anterior insula (Chua et al., related to disappointment [60]); (Red): striatum (Bartra et al., involved with emotional negative processing [46]).

Activity in the a priori amygdala (ROI), a brain area related to incongruent processing, during the viewing of messages exposing PSOE corruption by its sympathizers can be associated with a great degree of reliability of party closeness change from pre- to 30 days-post scan (r = 0.73, *p* = 0.017; F = 9.109). Activation in the anterior insula ROI, an area involved with disappointment, was positively and significantly correlated to party closeness change (r = 0.64, *p* = 0.043; F = 5.754). Finally, the striatum, a brain associated with emotional negative processing, also revealed a positive significant correlation to party closeness change among PSOE sympathizers (r = 0.813, *p* = 0.004; F = 15.617). Activity in the caudate ROI, which is related to risky processing, although positive, is quasi significant and associated with a change in party closeness (r = 0.568, *p* = 0.08; F = 2.15). For a complete listing of the Wilcoxon T values and complementary whole-brain analyses, see Figure 2 and Table 1. None of the ROI revealed links with the slight reduction of party closeness identified among PP sympathizers.

Interestingly, we then tested in a model whether the above-mentioned brain regions even predicted subsequent party closeness change after 30 days of receiving political information about the participants’ own party. By running a linear regression analysis, we found that only a model that includes the striatum as an independent variable (β = 3.983; F = 15.617; *p* = 0.004) was able to predict the reduction of party closeness in liberal sympathizers, and explained almost 62% of the variation in their party closeness (model R2 = 0.619). The power analysis of this regression model, with an effect size = 0.78, was 97%.

## 4. Discussion

Citizens are deeply worried about corruption [61]. The findings of surveys revealed that three quarters (76%) of Europeans considered that political corruption is widespread and over two thirds (68%) believed that the level of corruption in their country has increased over the past three years. Despite such concerns, advertising literature has not identified consistent results as to the effects of messages exposing corruption on party sympathy or on the psychological origin of their effects on the electorate. This is the first study that resorts to neuroimaging tools to examine the psychological origin of party closeness immobilism or change among sympathizers of Spain’s liberal (PSOE) and conservative party (PP) after exposure to a month of corruption messages linked to their party of preference. The behavioral findings indicate that only sympathizers of the liberal party revealed a significant decrease in party closeness after exposure to the campaign of corruption messages. Brain data provide some explanations as to the origin of this reduction as activities in areas linked to negative feelings, disappointment and self-relevance were connected to a great degree of reduction of party closeness over the course of one month. None of the hypothesized brain regions served to predict reduction of party closeness among PP sympathizers.

As regards the self-report responses, the findings of this study infer that campaigns exposing corruption had a greater effect on liberal (PSOE) sympathizers than their conservative (PP) counterparts. Leftist political sympathizers, in fact, reported less support to their party after viewing news items unveiling corruption. These findings line up with evidence of preceding studies suggesting that liberal individuals tend to be more autocritical and intolerant, and to prioritize social justice and equality. Supporters of the political right, in turn, reveal more sensitivity to threats, prioritize the status quo and seem to be more tolerant of inequality [29,62]. Together, these results highlight that intrinsic liberal and conservative values are key to the perception and level of persuasion of messages exposing corruption. The more liberal individuals report to be, the higher the level of degree of influence by messages revealing corruption of their party. Previous research has indeed supported this reasoning. An example is the study of de Figueiredo et al. [36] that concluded that exposing the criminal record of center-right candidates had no effect on the vote of their sympathizers of this political group. Riera et al. [37] also identified that politically involved liberal voters of the PSOE were, on the contrary, more diligent in punishing corrupt politicians (both liberal and conservative).

Neuroimaging methods such as functional magnetic resonance imaging (fMRI) are in a unique position to assess processes that are introspectively opaque. This technique offers the added advantage that neural responses can be recorded at the precise moment when the participant is subject to the stimuli [63] and therefore serve as a basis to predict behavior and changes in attitude. The current study, along these lines, is the first to reveal the psychological basis of the change in party closeness among liberal sympathizers when subject to advertising campaigns exposing corruption in their own party. The use of an a priori ROI approach yielded the finding that activations in the amygdala, anterior insula and striatum are significantly related to persuasion-induced changes in party closeness among PSOE sympathizers over the course of one month. In other words, PSOE sympathizers that reduced to a great extent their party closeness after one month of exposure to news of corruption revealed stronger activation in several preselected brain regions, namely the amygdala, striatum (this brain area even was able to predict over 60% of party closeness change) and anterior insula. The caudate, in turn, was only quasisignificant.

Activation of the amygdala during decision making processes encodes values of emotionally negative stimuli [64], fear [48] and disgust [65]. The findings of the current study are compelling in that the same portion of the amygdala that Westen [44] activated when voters processed incongruent information about their preferred political candidate served to predict a decrease in party closeness among PSOE sympathizers. These findings were reinforced by the capacity of the striatum to predict as this brain area is involved in detecting negative effects of the penalty domain [46] and in predicting behavioral and attitudinal changes [66]. The anterior insula, a brain area that responds to disappointment [49] and arousal [50] in decision-making environments, also served to predict a decrease in party closeness. Accordingly, PSOE sympathizers diminished their closeness to their party because of the repulse and disappointment they experienced while viewing corruption messages. Social psychology literature has associated these negative and disappointing feelings with depressive symptoms such as sadness, worthlessness and fatigue [67], which may have affected the reduction of sympathy by liberals. In fact, these neuropsychological functions have been largely involved with justice mechanisms, that is, the imposition of proportionate punishment [68,69]. Accordingly, the reduction in sympathy from liberals to their preferred party may have been rooted in cognitive processes involved with justice and punishment.

Taken together, the findings reflect the effectiveness of the negative and disappointing psychological mechanisms elicited by communication campaigns to shape party sympathy, thus supporting the findings of previous investigations emphasizing the usefulness of campaigns exposing corruption in provoking repulse in the electorate by lowering the level of trust and confidence in politicians [12].

In addition to the expected regions of interest (amygdala, striatum and anterior insula), the whole-brain analysis carried out on liberal sympathizers also revealed correlations between a change in party closeness and neural activity in the middle frontal gyrus, a brain region that predicts behavior change after exposure to persuasive messages and exhibits coactivation when individuals perceive the relevance of powerful messages [70,71]. Activation of areas linked to the self provides an integrative framework that promotes elaboration, organization of encoded information and assists in the choice of which motivational and behavioral representations guide behavior [60]. These results suggest that activations of the middle frontal gyrus may represent psychological responses to persuasive communications that index future behavior while being either consciously inaccessible or not captured by summary conscious reports following exposure to political messages. This notion is consistent with prior research on the neural basis of persuasion [63].

The current findings theoretically contribute to the literature challenging the effects of political advertising exposing corruption in reducing party closeness. Particularly, the paper contributes to a debate about whether voters care about political corruption, which is relevant to broader questions about electoral accountability and what kinds of indiscretion or misconduct might motivate voters to “throw the rascals out”. Previous research along this line has come to inconsistent conclusions as to the effects on the electorate when subject to information about corruption [13,43,72,73]. This paper differs from Eggers [28,74] and much of that literature because it does not use voting behavior as the dependent variable, but rather a measure of party closeness based on self-reporting, and it focuses particularly on party supporters rather than the electorate as a whole. More importantly, it seeks to contribute to our understanding of how and why party closeness might be affected by political corruption advertising, by using neuroimaging techniques to identify which types of feelings are associated with the change.

By considering the psychological differences between liberals and conservatives, and the biases derived from conscious self-reports, the current study advances findings gleaned from analyses carried out with behavioral and neuroimaging tools that fill the research gap. The study specifically proposes the notion that messages exposing the corruption of a political party are key to party closeness among sympathizers and exert an influence only among the liberal electorate. These conclusions are in line with those of previous political research that individuals of liberal tendency urge more punishment in response to corruption (regardless of the political party) and therefore may have a weaker partisan bias when compared to individuals with conservative tendencies [12,13,43]. This study thus confirms this reasoning at both neural and behavioral levels and supports the notion of the importance of reception of political information as an antecedent to create party affiliation and voting behavior [75].

This paper also constitutes an advance in the political communication field, as understanding the psychological bases of activity in the brain regions during persuasion may help update the substance of persuasion models [76], by considering, for example, the potential role of self-reference or implicit valuation. It also constitutes a step forward in the application of neuroimaging tools to gain insight into the physiological processes involved in political judgment and behavior, and in the literature on the motivated reasoning processes in the political environment. Previous research has explored the origin of political beliefs [77], partisan bias [78,79] and the modulating role of personality and ideological attitude on voting behavior [80]. This study, by contrast, takes a step forward as it resorts to neuroscience to arrive at the conclusion that psychological processes involved with self-relevance, disappointment and repulse are responsible for the reduction of closeness among liberal sympathizers exposed to campaigns unveiling corruption. In addition, this research advances in the evaluation of the neural mechanisms that determine human decision-making in general, and the user/consumer/citizen in particular [81].

The findings of this paper offer empirical evidence for the first time that messages exposing corruption of liberal (as opposed to conservative) political parties exerted an influence on the closeness of sympathizers as they activated psychological mechanisms linked to repulsion, disappointment and self-relevance. These conclusions, in line with those of Anduiza et al. [43], should lead liberal parties (e.g., the PSOE) to invest greater effort in assuring honest practices, avoid negative information (such as the recently widespread fake news) and attempt to dispel smear campaigns carried out by opposing parties. Liberal (vs. conservative) voters appear to forgive corruption in their party to a lesser degree as evidenced with their tendency to experience feelings of disappointment, reduction of support and confidence, and even voting avoidance [75]. The results of this study therefore indicate liberal candidates should also encourage arguing and disputing accusations of corruption if they want to avoid losing party identifiers.

Moreover, the immobilism in party closeness on the part of conservative voters should advise liberal parties not to spend excessive resources in spreading news of corruption about conservative parties, as they appear to have little effect on the conservative electorate. The results are also potentially of great interest to conservative parties as they could lead to the design of political campaigns with an emphasis placed on the negative practices of opposite parties. In doing so, they may induce a reduction in the level of sentiments of inclusion and affiliation among their opponents, and even diminish their level of support.

It must be noted that this study limited itself to measuring self-reported party closeness changes and not actual voting behavior. Although it is widely demonstrated that changes in party closeness are linked to variations in voting behavior [82,83], future political research should attempt to corroborate neural responses to corruption campaigns with real-life voting behavior by following the reasoning of [62,84]. Following the evidence showing that the partisan reasoning exerts the greatest influence when subjects confronting to contradictory information, this study resorted to neural responses to predict changes in party closeness on the basis of campaigns exposing corruption of the preferred party of participants. Future research should corroborate these findings by also measuring variations of support among opposite parties. Verification of the current findings requires further research in the framework of a broader range of European (and international) countries. Future studies should also corroborate if there are neural differences between liberals and conservatives during the processing of alternate corruption conditions involving, for example, corporations or families. The post scanning data were obtained one month following the fMRI session. Prospect studies should go deeper into the effect of time course (e.g., short vs. long term) in the neural mechanisms underlying the decline in party closeness. Finally, future research is in a position to assess for psychiatric and regarding emotional stability of voters when exposed to political campaigns.

Despite these potential caveats, this is the first study that applies a multimethodological approach to predict changes in party closeness derived from a one-month campaign exposing corruption, thus revealing days in advance the psychological mechanisms by which political communication affects political affiliation and even changes in voting behavior.

## Figures and Tables

**Table 1:** Brain regions resulting from the small volume correction (e.g., SVC, whole-brain with mask) and whole-brain (without mask) analyses associated with party closeness change among Spanish Socialist Workers’ Party (PSOE) sympathizers.

Contrasts and Regions	Peak MNI Coordinates (mm)			Cluster Size	T	Z	Effect Size	Study
	x	y	z					
PSOE corruption messages								
SVC analysis								
Striatum	−16	4	−4	8	5.37	3.40	1.07	Bartra et al. [46]
Caudate	−12	18	−3	2	5.67	3.50	1.10	Krain et al. [51]
Anterior insula	−30	3	9	2	5.15	3.33	1.05	Chua et al. [49]
Amygdala	−22	−4	−12	4	5.37	3.40	1.07	Westen et al. [44]
Whole-brain analysis								
Middle frontal gyrus	−22	49	19	8	6.01	3.60	1.13	

Effect Size = Z/√N (Smeets et al. [59]).

## Data Availability

The data presented in this study are available on request from the corresponding author.

## Appendix A

Only participants who did not suffer from the following medical issues were selected: vision problems, eye operations, fMRI problems, pregnancy, heart pacemaker, bone growth stimulator, heart valves, surgical staples, prosthesis (ear, knee, breast, penis and eyes), wire mesh implant, removable dental bridges, intrauterine devices, internal electrodes, neurostimulation systems, skin patch, breathing or movement problem and claustrophobia.
